# Simultaneous electro-generation/polymerization of Cu nanocluster embedded conductive poly(2,2′:5′,2′′-terthiophene) films at micro and macro liquid/liquid interfaces

**DOI:** 10.1038/s41598-023-28391-9

**Published:** 2023-01-21

**Authors:** Reza Moshrefi, Hanna Przybyła, Talia Jane Stockmann

**Affiliations:** Chemistry Department, Core Science Facility, Memorial University of Newfoundland, 45 Artic Ave., St. John’s, NL A1C 5S7 Canada

**Keywords:** Energy science and technology, Chemistry, Catalysis, Electrochemistry, Materials chemistry

## Abstract

Cu nanoparticles (NPs) have been shown to be excellent electrocatalysts, particularly for CO_2_ reduction – a critical reaction for sequestering anthropogenic, atmospheric carbon. Herein, the micro interface between two immiscible electrolyte solutions (ITIES) is exploited for the simultaneous electropolymerization of 2,2′:5′,2′′-terthiophene (TT) and reduction of Cu^2+^ to Cu nanoparticles (NPs) generating a flexible electrocatalytic composite electrode material. TT acts as an electron donor in 1,2-dichloroethane (DCE) through heterogeneous electron transfer across the water|DCE (w|DCE) interface to CuSO_4_ dissolved in water. The nanocomposite formation process was probed using cyclic voltammetry as well as electrochemical impedance spectroscopy (EIS). CV and EIS data show that the film forms quickly; however, the interfacial reaction is not spontaneous and does not proceed without an applied potential. At high [TT] the heterogeneous electron transfer wave was recorded voltammetrically but not at low [TT]. However, probing the edge of the polarizable potential window was found to be sufficient to initiate electrogeneration/electropolymerization. SEM and TEM were used to image and analyze the final Cu NP/poly-TT composites and it was discovered that there is a concomitant decrease in NP size with increasing [TT]. Preliminary electrocatalysis results at a nanocomposite modified large glassy carbon electrode saw a > 2 × increase in CO_2_ reduction currents versus an unmodified electrode. These data suggest that this strategy is a promising means of generating electrocatalytic materials for carbon capture. However, films electrosynthesized at a micro and ~ 1 mm ITIES demonstrated poor reusability.

## Introduction

Owing to their flexibility^1–3^ and biocompatibility^4^, conductive polymeric thin films have seen a significant rise in usage and interest. Motivated to reduce production cost, less expensive and involved preparation methods are being sought. For example, many polymerization methods generate relatively high average molecular weight materials that are robust; however, require electrodeposition onto an anode^5^, often binding the polymer to the electrode surface, or uses specialized bulky methods such as electrospinning^6^. In the case of the former, this likely eliminates the possibility of obtaining a free-standing film/conductive polymer electrode as the polymer is difficult to liberate from the anode and thus can limit the type of applications.

Meanwhile, metal nanoparticles (NPs) form the basis of numerous analytical and electrocatalytic platforms^7–9^; particularly copper (Cu) based NPs which are effective in catalyzing CO_2_ reduction^10–13^. Many metal NP preparation methods have emerged; however, the Brust-Schiffrin method, first described in 1994^14,15^, reproducibly generated low dispersity Au NPs by exploiting the interface between two immiscible electrolyte solutions (ITIES), i.e., the liquid|liquid interface. Indeed, the ITIES has recently come under increasing activity in the electrodeless synthesis of both metal NPs^15–27^ and conductive polymer films^4,28–36^. Initially, efforts were focused on the immiscible water|oil (w|o) interface^4,27–36^; however, these have recently expanded to the water|ionic liquid (w|IL)^21–26^ and oil|ionic liquid (o|IL)^20^ ones. In a simple 2-electrode configuration with one electrode immersed in either phase, the Galvani potential difference can be controlled externally via a potentiostat with the potential drop spanning 1–4 nm across the ITIES, *ϕ*_*w*_ – *ϕ*_*o*_ = $\Delta_{o}^{w} \phi$^8,37^.

Johans et al.^38^ were the first to describe an analytical solution for nucleation of metal NPs at the liquid|liquid interface. In their work, they emphasized the absence of defect sites that are common at a solid/solution interface; thus, there is a large thermodynamic barrier to particle formation at ITIES. Nevertheless, they^38^ and others^8,15–18,20,23,24,27,28,39,40^ were able to experimentally demonstrate electrochemically controlled metal NP nucleation at w|o interfaces. Interestingly, Nishi’s group has suggested that the molecular structure of the liquid|liquid interface is transcribed onto the NP framework^22^. They recently demonstrated that the w|IL interface played an important role mechanistically in the formation of nanostructures. Their IL was modified with a ferrocene (Fc) functional group making it redox active, and was exploited in the formation of Pd nanofiber arrays^22^.

Meanwhile, electropolymerization at liquid|liquid interfaces was initially investigated by Cunnane’s group^36^, and more recently by Scanlon’s group^4^ and ourselves^28^. In these later reports, large, free-standing polymer films were formed. In the case of Lehane et al.^4^ who generated PEDOT (poly(3,4-ethylenedioxythiophene)) using Ce^4+^ in aqueous and EDOT in *α*,*α*,*α*-trifluorotoluene (TFT), the films were shown to be highly stable and biocompatible. Our work showcased the simultaneous electropolymerization of 2,2′:5′,2′′-terthiophene (TT) and electrogeneration of Au NPs at a micro-ITIES (25 µm in diameter)^26^, building on Cunnane’s study at a large ITIES^29,30,32^. We demonstrated that miniaturization of the ITIES could be used to provide another layer of mechanistic and thermodynamic control towards smaller, low dispersity NPs.

Herein, this is expanded to the simultaneous electrogeneration and electropolymerization of Cu nanocluster incorporated poly-TT films. Electrochemical impedance spectroscopy was used to monitor film growth, while SEM and TEM imaging were used to compare film/NP morphology at different TT concentrations between the large and micro-ITIES. Two large ITIES platforms were investigated, including a 1.16 and 10 mm diameter interface. Initial testing of glassy carbon (GC) electrodes modified with the nanocomposite demonstrate excellent electrocatalytic activity towards CO_2_ reduction; however, films electrosynthesized at the 25 µm and 1.16 mm interfaces demonstrated poor stability and surface coverage.

## Methods

### Chemicals

Copper sulphate (CuSO_4_, > 98%), lithium sulphate (Li_2_SO_4_, > 98%), 1,2-dichloroethane (DCE, ≥ 99.0%), 1-bromooctane (99%), trioctylphosphine (97%), and 2,2′:5′,2′′-terthiophene (TT, 99%) were acquired from Sigma-Aldrich. All reagents were used without additional purification. Ultrapure water from a MilliQ filtration system (> 18.2 MΩ cm) was used throughout to generate aqueous solutions. The tetraoctylphosphonium tetrakis(pentafluorophenyl)borate (P_8888_TB) ionic liquid used as an oil phase supporting electrolyte was prepared as detailed previously^41^.

### Electrochemistry

**Figure 1:**
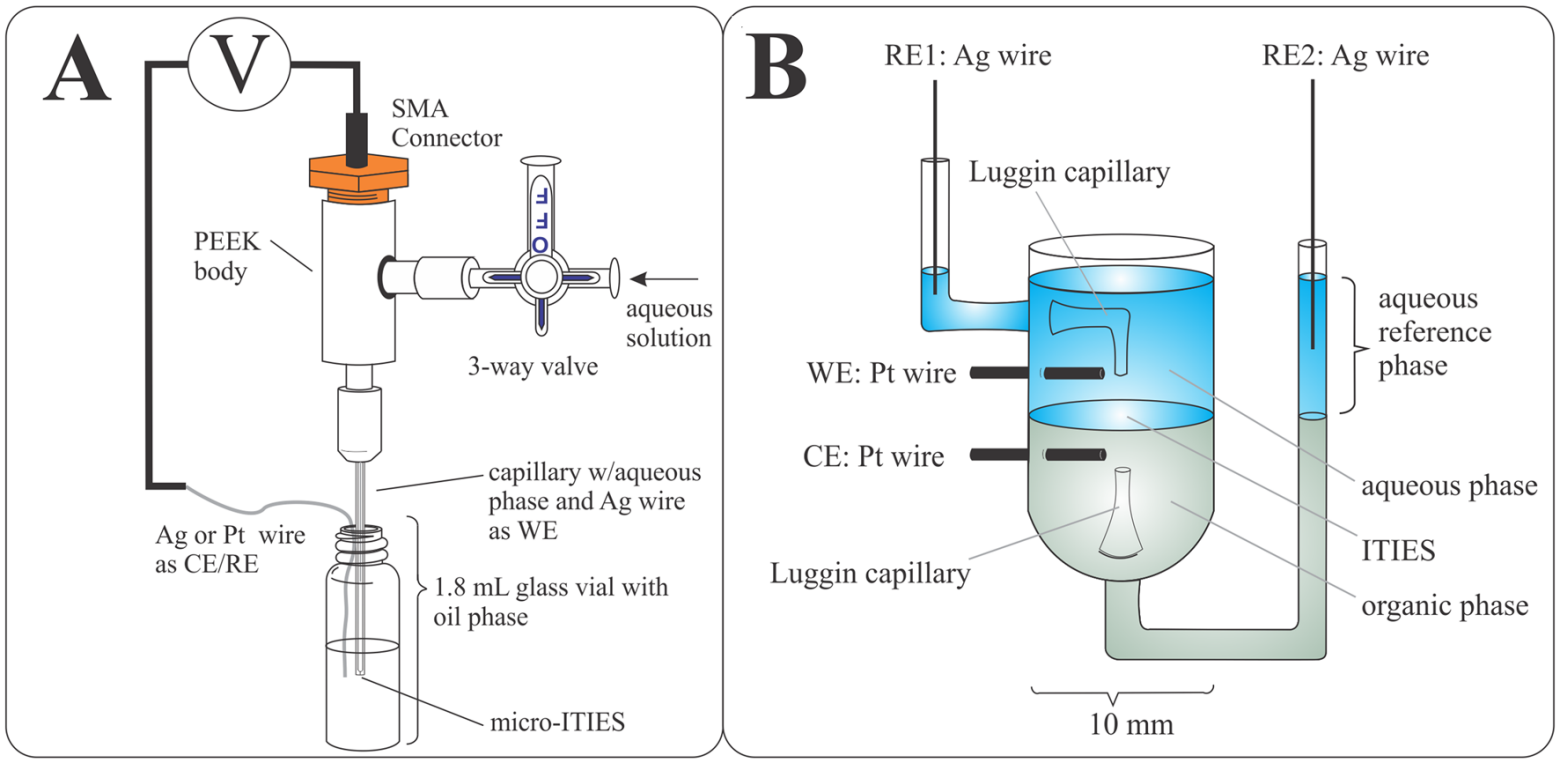
Diagram of the specialized (**A**) micro and (**B**) large ITIES (interface between two immiscible electrolyte solutions) cell. WE, CE, and RE refer to the working, counter and reference electrode leads, respectively.

Liquid|liquid electrochemical experiments were performed using a PG-618-USB potentiostat (HEKA Electroniks) in four-, three-, and two-electrode configurations at the large and micro-ITIES. In the 4-electrode mode a specialized 10 mm inner diameter cell with two Pt wires annealed into the side of the glass cell were connected to the working (WE) and counter electrode (CE) leads of the potentiostat as shown in Fig. 1B. The WE was positioned in the aqueous phase, while the CE was in the organic phase. Two references electrodes (RE) were also employed, one in either phase and inserted into Luggin capillaries built into the specialized cell with their tips facing each other ~ 5 mm apart with the liquid|liquid interface positioned in between them, see Fig. 1B.

**Scheme 1:**
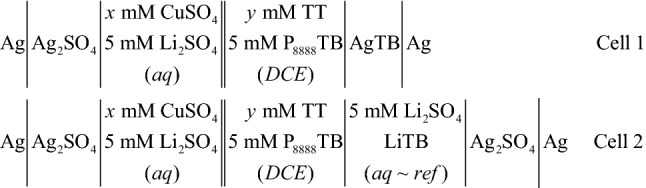
Electrolytic cells used where *x* mM of CuSO_4_ was added to the aqueous phase and *y* mM the electron donor, 2,2′:5′,2′′-terthiophene (TT), was added to the DCE phase in Cells 1 and 2, while 5 mM of the ionic liquid P_8888_TB (tetraoctylphosphonium tetrakis(pentafluorophenyl)borate) was employed as supporting electrolyte in the organic phase. The double bars indicate the polarizable potential w|DCE interface, with diameters of 25 µm in the case of the ITIES maintained at the tip of the micropipette (Cell 1), as well as 1.16 (Cell 1) or 10 mm (Cell 2) for the two large electrolytic cells, see Fig. 1.

When using the micro-ITIES in two-electrode mode, one Ag wire (Goodfellow Inc.) was integrated into the specialized pipette holder containing the aqueous phase and connected to the WE port of the head-stage, and another Ag wire immersed in the organic phase was connected to the CE/RE port, see Fig. 1A. The body of the holder was fabricated from poly(ether ether ketone) (PEEK). The pipette was backfilled with the aqueous phase through a syringe attached to a 3-way valve incorporated into the side of the specialized holder, then the tip of the pipette was immersed in the organic phase. The 25 µm diameter ITIES was maintained at the pipette tip using the syringe and monitored using an 18-megapixel CCD camera (AmScope) equipped with a 12 × magnifying lens assembly (Navitar). Micropipette fabrication has been described elsewhere^42^. Scheme 1 details the electrolytic cells employed. The experimental potential scale was referenced to the Galvani scale by simple SO_4_^2–^ transfer, whose formal ion transfer potential $\left( {\Delta_{o}^{w} \phi_{{{\text{SO}}_{4}^{2 - } }}^{o^{\prime}} } \right)$ was taken to be –0.540 V^16^.

A second large ITIES electrolytic cell (Cell 1, general configuration) was created by using the modified holder shown in Fig. 1A; however, an unmodified borosilicate capillary (2.0/1.16 mm outer/inner diameter, Sutter Instruments) was used in place of a micropipette. Additionally, a Pt wire counter and Ag wire reference electrodes were used, coupled to the integrated Ag wire as WE in the aqueous phase, in a 3-electrode configuration.

Electrochemical impedance spectroscopy (EIS) measurements were performed with a frequency range between 10 and 20 kHz, as well as a 20 mV peak-to-peak perturbation. EIS was only measured using Cell 1 at a 25 µm ITIES in a 2-electrode configuration.

Before or in-between experiments, electrolytic cells/capillaries were cleaned using the procedure outlined in the Supplementary Information (SI).

Electrocatalysis studies were performed using a 3-electrode cell connected to a CH Instruments potentiostat (model# CHI602E) with a glassy carbon (GC, Pine Research) WE (~ 4 mm in diameter) coupled with Ag/AgCl reference (Dek Research) and Pt wire counter electrodes.

### Transmission electron microscopy

All transmission electron microscopy (TEM) images were taken using the Tecnai Spirit transmission electron microscope with samples prepared on 200 Mesh Cu ultrathin/lacey carbon or 2 µm holey Au grids (Electron Microscopy Sciences).

### Scanning electron microscopy (SEM)

SEM imaging was performed using a JEOL JSM 7100 F equipped with energy dispersive x-ray (EDX) which were analyzed via DTSA II software provided by the National Institute of Standards and Technology (NIST) in the US, see https://www.nist.gov/services-resources/software/nist-dtsa-ii.

## Results and discussion

**Figure 2:**
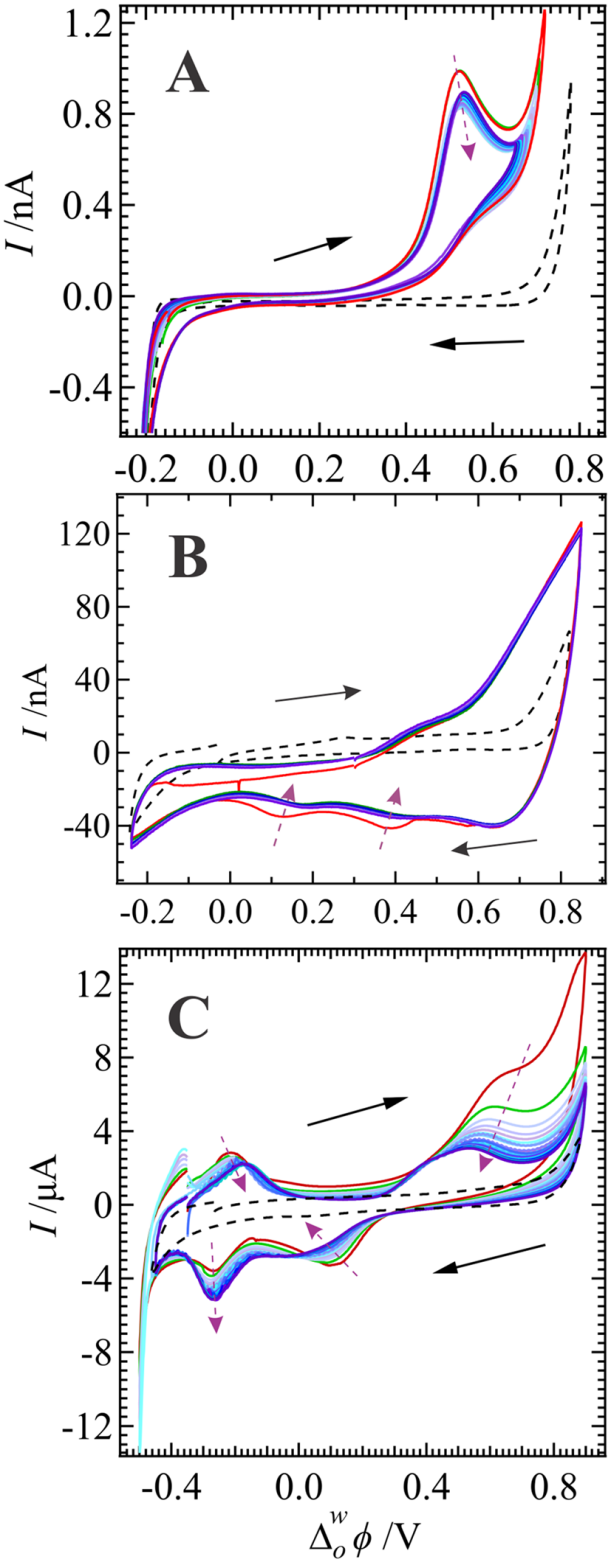
*i-V* curves recorded at 0.020 V s^–1^ using Cell 1 (**A**,**B**) and 2 (**C**), or the 25 µm, 1.16 mm, and 10 mm ITIES, respectively, with [TT] = 20 mM and [CuSO_4_] = 5 mM, while the black, dashed traces show the system without TT added, *i.e.*, blank curves. DCE was used as the organic solvent in all cases. Cyclic voltammograms have been scanned consecutively 25 times and overlaid such that the red and dark purple traces are the first and last scans, respectively; dashed, purple arrows indicate the evolution of the current signals with successive scans. Black arrows indicate scan direction.

The black, dashed curves shown in Fig. 2A-C illustrate cyclic voltammograms (CVs) recorded using Cells 1 and 2 at the micro (25 µm diameter), 1.16 mm, and ~ 10 mm diameter ITIES, respectively, without TT added, but with 5 mM of CuSO_4_ in the aqueous phase. In each case, the polarizable potential window (PPW) is limited by the transfer of the supporting electrolyte ions. The large positive current increase at positive potentials is owing to the transfer of Li^+^/Cu^2+^ from water to oil (w → o) or TB^–^ from o → w, while the sharp negative increase in current towards negative potentials is owing to the transfer of SO_4_^2–^ from w → o and P_8888_^+^ from o → w^8,43^.

The red, solid trace shows the initial *i-V* cycle at the micro-ITIES (Fig. 2A), after addition of 20 mM of TT to DCE. A positive peak-shaped wave was observed during the forward scan, towards positive potentials, with a peak potential (*E*_*p*_) at roughly 0.52 V. During the reverse scan, towards negative potentials, a sigmoidal wave was observed with a half-wave potential $\left( {\Delta_{o}^{w} \phi_{1/2} } \right)$ at ~ 0.515 V. Since the interface was maintained at the tip of a pulled micropipette, the diffusion regime inside and outside the pipette is asymmetric. The former behaves under linear diffusion owing to geometric confinement within the micropipette, while the latter is hemispherical with responses similar to an inlaid disc ultramicroelectrode (UME)^44^. Thus, this signal is consistent with the transfer of negative charge from o → w during the forward scan, such that the process is diffusion limited by a species in the aqueous phase. Therefore, it was hypothesized that this signal is owing to electron transfer from TT in DCE across the ITIES to Cu^2+^ in water; whereby, Cu^2+^ is reduced to Cu^0^ and forms nanoparticles, while TT is oxidized and electropolymerized.

To investigate this, the system was cycled a total of 25 times with these *i-V* curves overlaid in Fig. 2A; the dashed, purple arrow indicates how the peak current (*i*_*p*_) signal evolves with each consecutive scan. There is only a small change in *i*_*p*_ that may be owing to a localized consumption at the interface, or the formation of a nanoparticle incorporated polymer film at the ITIES. The latter would fundamentally alter the effective surface area of the interface as well as its charge transfer characteristics; both would impact the magnitude of *i*_*p*_. Cell 1 was also tested using [CuSO_4_] = 0 mM and [TT] = 20 mM; however, no peak-shaped signal was recorded (data not shown). Thus, it is likely that Li^+^ does not interact with TT and is a good electro-inactive supporting electrolyte. However, when [TT] was decreased to 10 and 5 mM in DCE, no electron transfer wave was recorded voltammetrically (data not shown).

In all cases at the micro-ITIES, an aqueous droplet was ejected onto a TEM grid for imaging using the syringe equipped on the back of the modified holder. Even at 5 and 10 mM TT, a film-like deposit was visible on the substrate under an optical microscope. Therefore, despite no observable electron transfer wave, cycling at the PPW edge-of-scan can induce electrogeneration of the nanocomposite film. These results agree well with our recent work at the w|DCE micro-interface using KAuCl_4_(aq) and TT(org)^28^; whereby, a thin conductive polymer film, with embedded Au NPs was formed. Lehane et al.^4^ also recently employed a large w|DCE interface in the electropolymerization of PEDOT with Ce^4+^ as oxidant/electron acceptor in the aqueous phase. Moreover, electrodeposition of Cu, Au, Pd, and Pt NPs at large^16,30,38,40,45^ and micro^27,39^ interfaces have been demonstrated previously using metallocenes as electron donors dissolved in oil.

An ITIES with a diameter of 1.16 mm using a 3-electrode configuration was also tested voltammetrically, see Fig. 2B. The overall CV profile is similar to the one at a ~ 10 mm ITIES (see below) with a signal at ~ 0.7 V which is likely the electron transfer wave; however, two negative current peaks were recorded at roughly 0.4 and 0.15 V during the scan from positive to negative potentials. These may be the re-oxidation of Cu^0^ or anion adsorption waves. Future work will focus on in situ spectroscopic methods to evaluate these two curve features.

Next, film generation was investigated at a 10 mm diameter ITIES using Cell 2 (see Fig. 2C). When scanning from negative to positive potentials, two peak-shaped waves were observed at – 0.22 and 0.66 V, while on the reverse scan towards negative potentials, two peak signals were recorded at 0.09 and – 0.26 V. The two peaks towards the negative end of the PPW form a reversible signal with a $\Delta_{o}^{w} \phi_{1/2}$ of – 0.240 V which may be the result of the adsorption of anions at the surface of the growing composite film. Indeed, this agrees well with the results of Lehane et al.^4^ The two signals at the positive end of the PPW are irreversible electron transfer similar to the result observed at the micro-ITIES in this potential region. With repeated sweeps of the potential, the irreversible electron transfer wave shifts to more negative potentials indicating a reduction in the required overpotential. As mentioned above, the liquid|liquid interface is initially free of nucleation sites which increases the amount of applied driving force necessary to achieve NP nucleation/polymerization^38^ versus at a solid/electrolyte one. However, once the film begins forming, the population of viable sites increases which is reflected in the concomitant decrease in peak potential of the electron transfer wave. Simultaneously, the signal decreases in current intensity which is likely owing to the consumption of material in the vicinity of the ITIES. The nanocomposite film formed in the large ITIES cell was thick, brittle, and difficult to extract from the cell. However, pieces were deposited onto glass substrates for SEM imaging and onto a GC electrode for electrocatalytic testing (see below).

The voltammetric negative peak during the reverse scan in Fig. 2C with a $\Delta_{o}^{w} \phi_{1/2}$ at – 0.240 V may be owing to the reorganization of the electric double layer (EDL) and adsorption of supporting electrolyte anions on either side of the forming liquid|solid|liquid interface^4^. As shown by Scanlon’s group^4^, supporting electrolyte anions from both phases stabilize film growth through adsorption; however, due to the size and shielded negative charge on B(C_6_F_5_)_4_^–^, it is likely a minor contributor and most anion adsorption comes from SO_4_^2–^ on the aqueous side. Moreover, SO_4_^2–^ is molecularly smaller with exposed dense negative charge on the oxygens, doping the film with sulphate is more efficient in stabilizing and neutralizing the film as it forms. However, during the later stages of film growth, diffusion of anions through the film is inhibited making doping/de-doping a slow process and causing voltammetric peak broadening^46^. Regardless, the film will likely be p-doped.

Both micro and large ITIES experiments were also performed at open circuit potential (OCP), i.e., without an applied external potential. In both cases, no film or NPs were observed. Thus, an applied potential is required to induce nanocomposite film formation.

**Figure 3:**
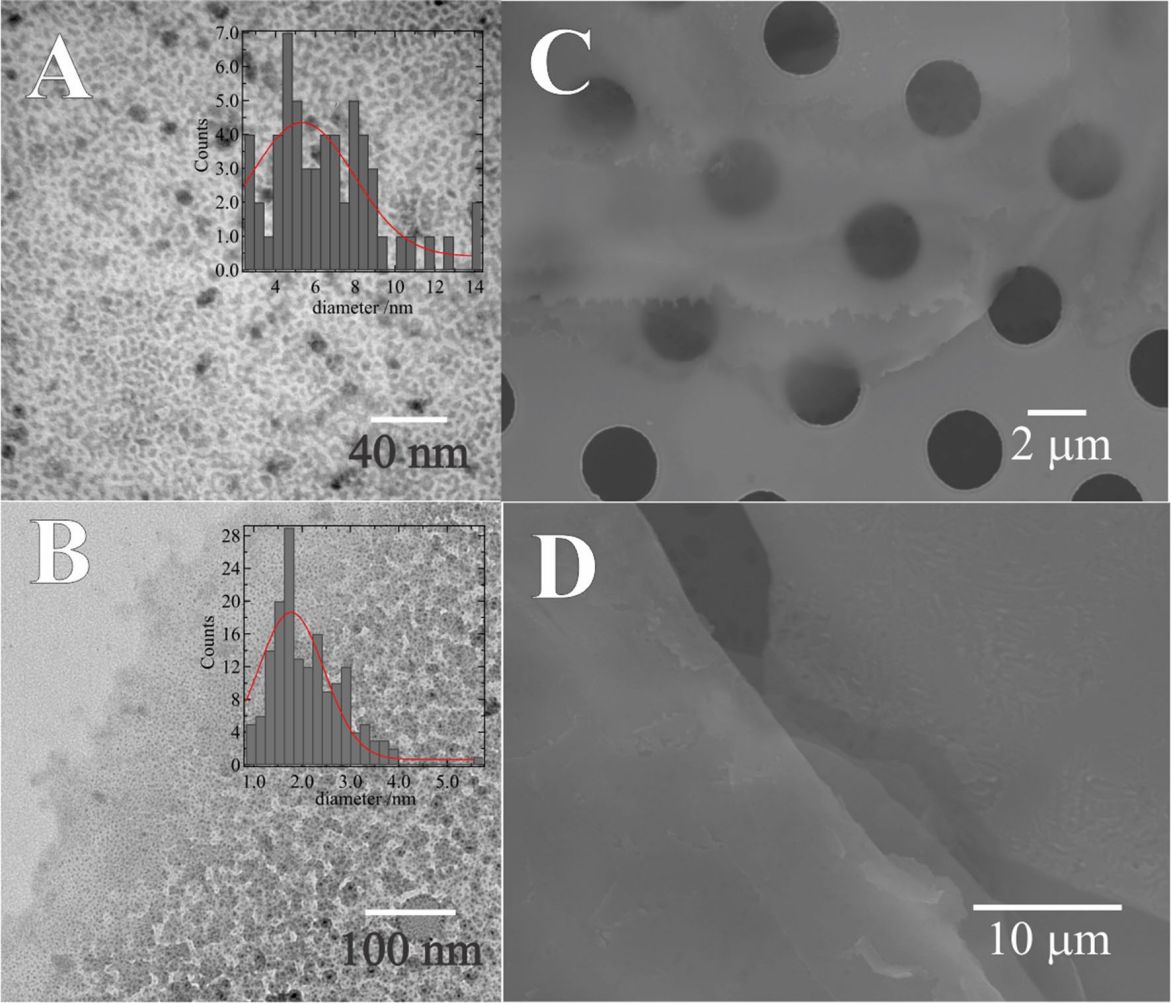
TEM micrographs obtained for aqueous phase samples after 25 consecutive CV scans using Cell 1 at a w|DCE interface with [CuSO_4_] = 5 mM, as well as 5 (**A**) and 20 mM (**B**) of TT in DCE and deposited on a 2 µm diameter holey Au TEM grid; inset are histograms of Cu NP/nanocluster sizes. (**C**) SEM micrograph of the sample shown in B. (**D**) SEM image of the film generated at a large ITIES using Cell 2 with 5 mM of CuSO_4_(aq) and 20 mM of TT(org).

Using the syringe affixed to the back of the pipette holder, a droplet of the aqueous phase was ejected from the tip of the micropipette after 25 consecutive CV scans using Cell 1 with 5 mM of CuSO_4_ paired with 5 or 20 mM of TT in DCE and deposited on a holey-Au TEM grid. Next, the TEM grids were imaged using both TEM and SEM (see Fig. 3). TEM micrographs in Fig. 3A and B show the low dispersity spherical Cu NPs electrogenerated and embedded within the poly-TT film with average sizes of 5.3 and 1.7 nm at [TT] equal to 5 and 20 mM, respectively. NP sizes were measured using ImageJ software and collated into histograms plotted inset in Fig. 3; average NP sizes were determined by curve fitting the histograms with a Gaussian distribution. Fig. S1 of the SI shows the histogram of Cu NP sizes with the poly-TT film formed using Cell 1 with 10 mM of TT in DCE. With increasing [TT] the NP size decreased such that at TT = 10 or 20 mM, Cu particles are in the range of nanoclusters, *i.e.*, 1.3 or 1.7 nm in diameter, respectively^47,48^. In this case, with higher [TT] the thermodynamics and kinetics of the heterogeneous electron transfer reaction are enhanced facilitating faster electropolymerization, which in turn likely limits the size of the Cu nanoclusters.

TEM micrographs were also obtained for films electrogenerated at the 1.16 and 10 mm interfaces and deposited onto 200 mesh Cu lacey carbon TEM grids. Fig. S3 of the SI shows the TEM micrographs along with histograms for the analysis of the Cu NP sizes performed using ImageJ software. Cu NPs at 1.16 and 10 mm ITIES demonstrate a high dispersity despite Gaussian curve fitting showing peaks at 2.2 and 4.1 nm. Errors shown inset in Fig. S3 are for the Gaussian peak position/fitting. Fig. S4 shows a photograph of the aqueous droplet suspended from the unmodified capillary (1.16 mm in diameter) post Cu NP/poly-TT electrogeneration and after removal from the oil phase. A thin-film can be observed spread across the droplet surface.

Figure 3C depicts the SEM micrograph of the nanocomposite Cu NP/poly-TT film deposited on a holey-Au TEM grid. The film was dense, compact, and smooth; however, it was also quite fragile and broke apart easily. Relatively large sections can be seen covering the TEM grid and occluding several of the 2 µm holes. These images agree well with the reported morphology for electropolymerized terthiophene in low current densities^49,50^ and are similar to the PEDOT film electropolymerized at a large ITIES reported by Scanlon’s group^4^. Cu NPs were confirmed by energy dispersive x-ray (EDX) spectroscopy performed during SEM imaging (data not shown). 24-h shake-flask experiments performed in a 2 mL vial (large ITIES) using the same electrolyte compositions and TT concentrations as Cell 2 revealed no observable thin film or Cu NPs. Therefore, while not strictly observable voltammetrically at [TT] = 5 mM, by probing the positive edge of the PPW one can initiate Cu NP/poly-TT electrodeposition at relatively low [TT]. Moreover, this can be achieved without the use of extreme overpotentials that risk over-oxidizing the film^31,51^.

Figure 3D shows the film generated at the large ITIES with [CuSO_4_] = 5 mM and [TT] = 20 mM after 1000 CV cycles. The film is smooth; however, the Cu NPs cannot be resolved with SEM. Fig. S2A in the SI shows the CVs recorded during Cu NP/poly-TT electrosynthesis with [CuSO_4_] = 1 mM and [TT] = 5 mM. The first and every subsequent fifth scan was plotted. The Cu^2+^/TT reduction/oxidation and Cu re-oxidation waves are visible at roughly 0.85 and 0.55 V, respectively. The Cu NP/poly-TT film was extracted from the cell and deposited on a glass slide, then imaged in the SEM. Fig. S2B shows the SEM micrograph, while Fig. S2C and D contain plots of the EDX spectra obtained at the two points indicated in Fig. S2B. In Fig. S2B, the EDX spectra show that point C is rich in Cu and likely an agglomeration of Cu NPs, while point D is the polymer film itself containing sulphur and carbon.

The stepwise nucleation, oligomerization, and elongation of complex polymer/composite materials at liquid|liquid interfaces has been described by Vignali et al.^31^, Robayo-Molina et al*.*^52^, and recently by us^28^. The early stages of TT electropolymerization via heterogeneous electron transfer and electrodeposition can be described generally by the following,

1 $${\text{Cu}}^{{{2} + }} \left( {{\text{aq}}} \right) \, + {\text{ H}} - {\text{TT}}\left( {{\text{org}}} \right) \, \to {\text{ Cu}}^{ + } \left( {{\text{aq}}} \right) \, + {\text{ H}} - {\text{TT}}^{ + \bullet } \left( {{\text{org}}} \right),$$

2 $${\text{Cu}}^{ + } \left( {{\text{aq}}} \right) \, + {\text{ H}} - {\text{TT}}\left( {{\text{org}}} \right) \, \to {\text{ Cu}}^{0} \left( {\text{s}} \right) \, + {\text{ H}} - {\text{TT}}^{ + \bullet } \left( {{\text{org}}} \right),$$

3 $${\text{2H}} - {\text{TT}}^{ + \bullet } \left( {{\text{org}}} \right) \, \to {\text{ H}} - {\text{TT}}^{ + } - {\text{TT}}^{ + } - {\text{H}}\left( {{\text{org}}} \right),$$

4 $${\text{H}} - {\text{TT}}^{ + } - {\text{TT}}^{ + } - {\text{H}}\left( {{\text{org}}} \right) \, + {\text{ 2OH}}^{-} \left( {{\text{aq}}} \right) \, \to {\text{ TT}}_{{2}} \left( {\text{s}} \right) \, + {\text{ H}}_{{2}} {\text{O}}\left( l \right).$$

where H-TT is the terthiophene molecule emphasizing the proton at the α- or β-carbon position on one of the terminal thiophene units, TT^+•^ is the radical cation, and TT_2_ is the dimer. This initial stage is likely thermodynamically prohibitive at the liquid|liquid interface since it lacks nucleation sites^38^. However, once seeded with Cu^0^ nuclei and the positively doped TT oligomers as capping agents, the thermodynamics likely greatly improve, as mentioned above. It should be emphasized that the glass walls of the micropipette likely behave as nucleation sites; this would also apply to the walls of large glass ITIES electrolytic cells. Using Eqs. (1–4) as a basis, an overall reaction can be composed,

5 $${\text{Cu}}^{{{2} + }} \left( {{\text{aq}}} \right) \, + {\text{ 2H}} - {\text{TT}}\left( {{\text{org}}} \right) \, + {\text{ 2OH}}^{-} \left( {{\text{aq}}} \right) \, \to {\text{ Cu}}^{0} \left( {\text{s}} \right) \, + {\text{ TT}}_{{2}} \left( {\text{s}} \right) \, + {\text{ H}}_{{2}} {\text{O}}\left( l \right),$$

The overall electron transfer potential $\left( {\Delta_{o}^{w} \phi_{{{\text{ET}}}} } \right)$ for Eq. (5) can be written as^27,28,38^,

6 $$\Delta_{o}^{w} \phi_{{{\text{ET}}}} \approx E_{{{\text{TT}}^{{ +\cdot }} {\text{/TT}}}}^{{o^{\prime},{\text{DCE}}}} - E_{{\text{Cu(II)/Cu}}}^{{o^{\prime},{\text{H}}_{{2}} {\text{O}}}} + \left( {0.059\;{\text{V}}} \right)\left( {14 - {\text{pH}}} \right),$$

where $E_{{{\text{TT}}^{{ +\cdot }} {\text{/TT}}}}^{{o^{\prime},{\text{DCE}}}}$ and $E_{{\text{Cu(II)/Cu}}}^{{o^{\prime},{\text{H}}_{{2}} {\text{O}}}}$ are the standard redox potentials for TT^+•^/TT and Cu^2+^/Cu^0^ and were taken to be 1.20^28^ and 0.342 V^53^, respectively. In this way, $\Delta_{o}^{w} \phi_{{{\text{ET}}}}$ was calculated to be 1.57, 1.36, and 1.18 V for pH’s 2, 5.5–6, and 8.5, respectively; since Δ*G* = $nF\Delta_{o}^{w} \phi_{{{\text{ET}}}}$^54^, this leads to Δ*G* >  > 0 that nevertheless decreases with increasing pH. These values are much higher than the experimentally determined values at pH ~ 5.5–6; thus, the difference is likely the thermodynamic contribution of the glass walls. However, silanization of the inside of the micropipette resulted in no observable change in the film produced (data not shown).

**Figure 4:**
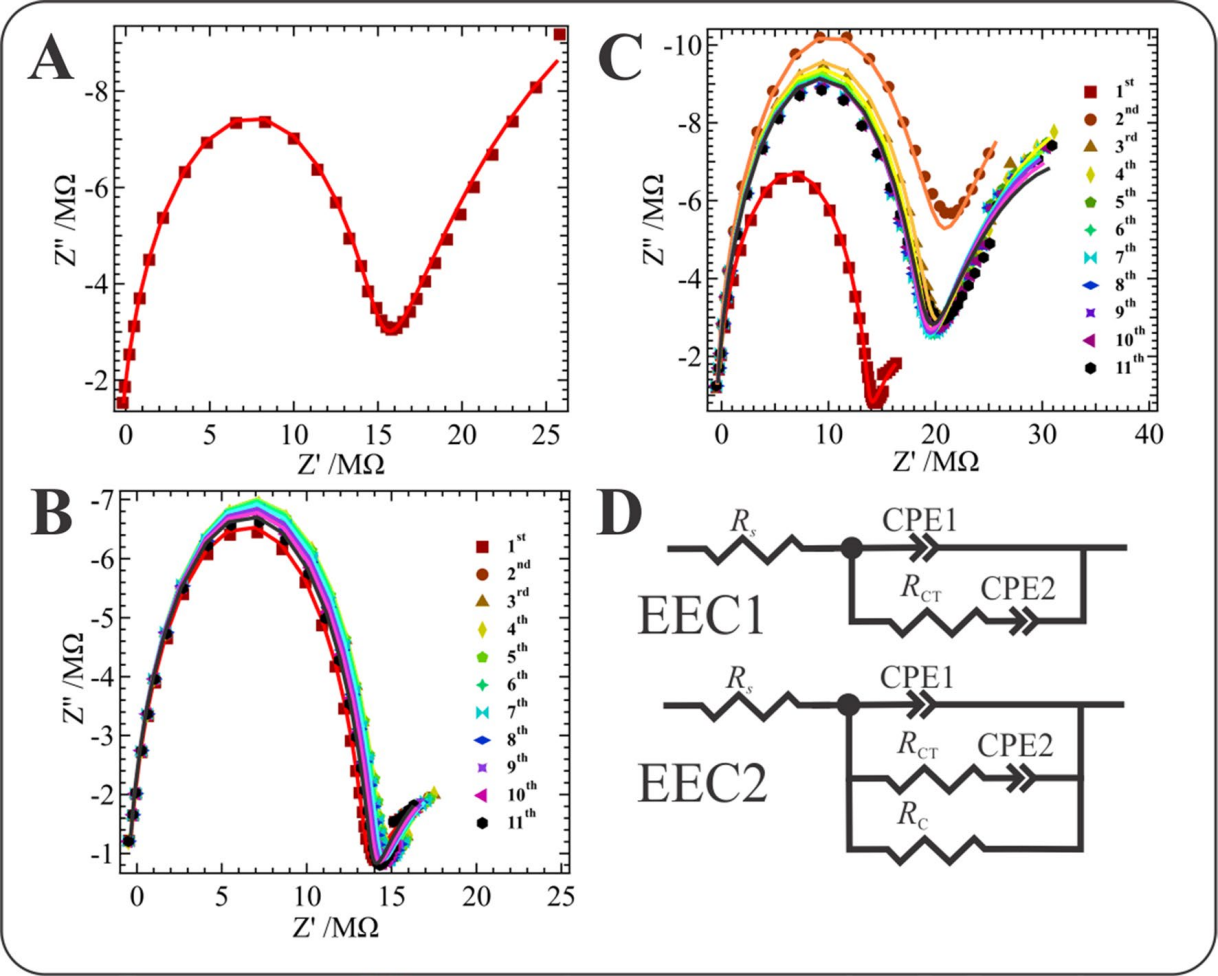
The measured (markers) and the fitted (solid line) Nyquist diagrams acquired using Cell 1 with 0 (**A**), 5 (**B**), and 20 mM (**C**) of TT added to the DCE phase as well as 5 mM of CuSO_4_(aq). Spectra were obtained with a direct applied potential (*V*_DC_) of ~ 0.7 V after performing one CV cycle using the potential range shown in Fig. 2 with *v* = 0.020 V s^–1^; similarly, in-between each spectrum in B and C a CV pulse was applied. (**D**) equivalent electric circuits (EEC) for a simple ion transfer (EEC1) or coupled electron and ion transfer during electro-generation (EEC2) of the thin film at an ITIES, such that *R*_*s*_, *R*_CT_, and *R*_*C*_, are the solution, charge transfer, and kinetic resistance, while CPE1 and CPE2 are constant phase elements.

Next, electrochemical impedance spectroscopy (EIS) was employed during electrosynthesis at the micro-ITIES to elucidate the underlying physical and electrochemical dynamics of film formation. The sinusoidal applied potential waveform (*V*_AC_) can be described by^55^,

7 $$V_{{{\text{AC}}}} \left( {\omega t} \right) = V_{DC} + V_{0} \sin \left( {\omega t} \right),$$

where *V*_DC_ and *V*_0_ are the applied DC voltage and AC voltage amplitude (0.020 V peak-to-peak), respectively, while *ω* (= 2*πf*) is the angular frequency and *t* is time. In each case, *V*_DC_ of roughly 0.7 V was applied (*vs.* $\Delta_{o}^{w} \phi_{{{\text{SO}}_{4}^{2 - } }}^{o^{\prime}}$) and a CV was performed between each impedance measurement at a scan rate of 0.020 V s^–1^. In this way, the Nyquist diagrams in Fig. 4A–C were recorded using Cell 1 at a 25 µm diameter interface with [TT] equal to 0, 5, and 20 mM in DCE, respectively. The semi-circle at high-frequency and partial semi-circle at low-frequencies describe the two typical branches of impedance spectra that are associated with electrical and electrochemical dynamics (i.e., mass transport), respectively^55^. In the case of the liquid|liquid interface, the high-frequency region is often associated with the capacitance of the back-to-back EDLs found in either phase, while the low frequency region is influenced by ion diffusion and electron transfer reactions^55–57^. These two features give rise to two “time constants” ^55^, so called since they are often modelled in equivalent circuits using a resistor  and capacitor (*C*_*╧*_) in parallel.

Equivalent electric circuits (EECs) used to model the impedance data have been drawn in Fig. 4D, which include constant phase elements (CPEs) in place of simple capacitors, in parallel with resistors added to model charge transfer reactions (*R*_CT_) and kinetic resistance (*R*_*C*_). As is common, a resistor was added in series to account for the total solution resistance (*R*_*s*_). EEC1, a Randles-like equivalent circuit, and EEC2 feature the two terminals at either end for the WE and CE/RE employed experimentally. The 2-electrode configuration limits parasitic impedance artifacts from cabling and/or the CE and RE that are often observed at 3- and 4-electrode cells^57,58^. Operating at the micro-ITIES has added benefits and is able to resolve the charge transfer resistance over the solution electrolyte resistance; moreover, by repeatedly using the same micropipette, one can greatly enhance reproducibility.

EIS offers avenues to valuable physical insight into charge transfer processes at the liquid|liquid interface. The geometric capacitance (*C*_*geo*_), recently modeled by von Hauff and Klotz^55^ in the context of perovskite solar cells, may be considered analogous to the structural parasitic coupling elements proposed and modelled by Trojánek et al.^57^ for a 4-electrode cell, which they modelled using a 4-terminal EEC. In either case, *C*_*geo*_ is normally modelled in parallel with all the other circuit elements. If it exceeded the individual capacitive circuit elements, i.e., CPE1 and CPE2 in Fig. 4D, then the high-frequency semi-circle would become enlarged and dominate the impedance spectrum. However, by operating in the 2-electrode mode, and ensuring that the overall impedance of the micropipette was < 1 MΩ during single phase experiments, then individual circuit elements can be resolved^59^. Thus, *C*_*geo*_/parasitic coupling elements can be ignored, greatly simplifying EEC modelling^57,60,61^. The faradaic impedance (*Z*_*f*_) is described by^57^,

8 $$Z_{f} = R_{{{\text{CT}}}} + Z_{W} = \frac{RT}{{z^{2} F^{2} Ak_{f} c_{i,w}^{*} }} + \left( {1 - j} \right)\sigma \omega^{{ - {\raise0.5ex\hbox{$\scriptstyle 1$} \kern-0.1em/\kern-0.15em \lower0.25ex\hbox{$\scriptstyle 2$}}}} ,$$

where *k*_*f*_ is the apparent rate of charge transfer, *A* is the surface area of the interface, *F* is Faraday’s constant (96,485.33 C mol^–1^), *R* is the gas constant (8.314 J mol^–1^ K^–1^), *T* is the absolute temperature (298.15 K), *z* is the number of electrons transferred, and *Z*_*W*_ is a Warburg impedance, within which, *j*^2^ = –1, and *σ* is defined as^57^,

9 $$\sigma = \frac{RT}{{2^{{{\raise0.5ex\hbox{$\scriptstyle 1$} \kern-0.1em/\kern-0.15em \lower0.25ex\hbox{$\scriptstyle 2$}}}} z^{2} F^{2} A}}\left[ {\frac{1}{{D_{i,o}^{{{\raise0.5ex\hbox{$\scriptstyle 1$} \kern-0.1em/\kern-0.15em \lower0.25ex\hbox{$\scriptstyle 2$}}}} c_{i,o}^{*} }} + \frac{1}{{D_{i,w}^{{{\raise0.5ex\hbox{$\scriptstyle 1$} \kern-0.1em/\kern-0.15em \lower0.25ex\hbox{$\scriptstyle 2$}}}} c_{i,w}^{*} }}} \right].$$

Here, *D*_*i,o*_ and $c_{i,o}^{*}$ are the diffusion coefficient and bulk concentration of species *i* in the oil (o) phase, while subscript w indicates their values in the water phase. CPEs were used to account for the dynamic nature of the interface and facilitate the evolving properties of the growing polymer-NP network. CPE impedance can be written generally as^62^,

10 $$Z\left( \omega \right) \approx \frac{1}{Q}\left( {j\omega } \right)^{ - n} .$$

In which *Q* (F s^1–*n*^) is a constant and when *n* = 1 the element is a perfect capacitor, while *n* = 0.5 is in line with a typical Warburg element for semi-infinite diffusion.

At a freshly cleaned micro-ITIES, a CV was performed followed by impedance measurement using a *V*_DC_ = 0.7 V; whereby, the CV-EIS pulse sequence was performed a total of 11 times. Figure 4A shows the impedance spectra using Cell 1 with [TT] = 0 and [CuSO_4_] = 5 mM, i.e., a blank spectrum. The pronounced low-frequency tail is likely owing to the relatively high *V*_DC_ close to the edge of the PPW where supporting electrolyte ions will undergo transfer, e.g., Li^+^ w → o. This spectrum agrees well with one shown recently by Mareček’s group^59^ which was associated with simple tetraethylammonium ion transfer at a micro-ITIES.

At low [TT] (Fig. 4B) the impedance profile does not undergo significant change; however, the tail in the low-frequency region is greatly suppressed relative to the blank spectra performed in the absence of TT (Fig. 4A). This may indicate that the film has formed after the first CV-EIS sequence and is blocking, or at least inhibiting simple ion transfer of the supporting electrolyte.

Figure 4C shows the response at high [TT] in which the high frequency region of the EIS increases by 50% between the first and second CV-EIS pulse sequence. There is then a small decrease in the high frequency branch, which stabilizes across the 3rd to 11th iteration. Meanwhile, the low-frequency branch at high [TT] becomes more pronounced with each iteration. Based on the CV results (Fig. 2A), the film forms immediately generating a liquid|solid|liquid interface and, thus, the low-frequency branch is then associated with mediated electron transfer between Cu^2+^(aq) and TT(org) rather than simple ion transfer. Thus, as the film grows the electron transfer properties change (see below).

Nevertheless, these data indicate a change in the nature of the interface brought about by the development of a liquid|solid|liquid system that occurs immediately upon application of the first CV-EIS. It has been shown that forming a barrier at the interface mainly affects the low-frequency region^28^. However, as the film develops, and likely due to the build up of local micro-convections in this domain^63^, it was found that by pushing the impedance measurement to lower and lower frequencies the interface becomes unstable. This either lead to a higher noise level or the interface itself physically broke down, erupting into the organic phase as an electrophoretically induced droplet. Thus, it was not possible to carry out EIS measurements below 10 Hz.

**Figure 5:**
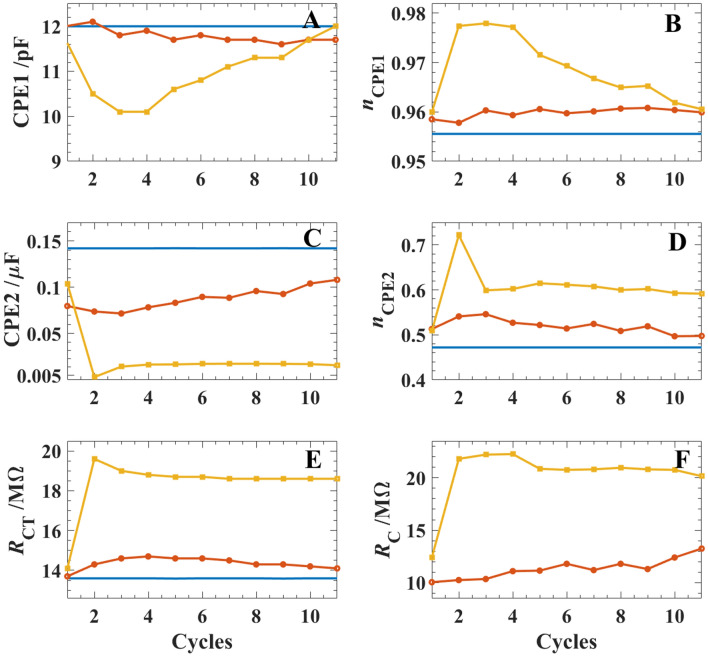
Equivalent electric circuit (EEC) parameter values obtained from fitting experimental impedance spectra from Fig. 4A–C using EEC2 depicted in Fig. 4D, with the orange filled circle and yellow filled square, curves derived from curve fitting impedance data obtained from Cell 1 with [TT] = 5 and 20 mM, respectively. The blue trace was determined using Cell 1 with [TT] = 0 mM.

EEC2 (Fig. 4D) was employed during thin film growth in the presence of CuSO_4_(aq) and TT(org), while EEC1 was used in the absence of TT, see the blue, solid curves in Fig. 5. Figure 5 shows the changes in the six EEC parameters after each CV-EIS iteration. With 5 mM of TT in the system, orange filled circle curves, CPE1 shows little deviation from the initial value of ~ 12.0 pF with only a slight decrease over the 11 CV-EIS pulse sequences before finally stabilizing at ~ 11.7 pF. As mentioned above, this circuit element is typically associated with the liquid|liquid back-to-back EDLs and these small changes in capacitance may be owing to ion rearrangement on either side of the interface during thin film electrogeneration or changes in the surface morphology at either side of the liquid|solid|liquid junction. At [TT] = 20 mM, yellow filled square traces in Fig. 5, there is a sudden decrease in CPE1 until the 4th CV-EIS pulse sequence, after which the element recovers and increases to a final value of 12.1 pF. In both cases, *n* varies between roughly 0.96 and 0.98, indicative of a conventional capacitor with likely an inhomogeneity in ion distribution at the interface.

The *n* value for CPE2, *R*_CT_, and *R*_*C*_ increase slightly with [TT] = 5 mM and each CV-EIS pulse iteration, while all three parameters show a much larger increase at [TT] = 20 mM. *R*_CT_ and *R*_*C*_ are ½ and 2 × as high, respectively, when [TT] = 20 mM versus 5 mM. This agrees well with impeded electron transfer or ion diffusion through the growing polymer/NP network. The limited diffusion within the nanocomposite structure likely results in limited anion exchange to neutralize individual TT units contributing to its p-doped nature and accumulation of negative charge on the surface of the Cu NP/poly-TT composite. These values agree with those recently reported by us for Au NP/poly-TT nanocomposites electrogenerated at a micro-ITIES^28^ after multiple CV-EIS pulses were performed. Herein, the interface was monitored in situ using a CCD camera equipped with a 12 × zoom lens assembly with a 10–12 cm working distance; however, unlike the Au NP/poly-TT film grown previously^28^, the Cu NP/poly-TT nanocomposite film was clear/colourless and, therefore, no observable change was observed optically.

**Figure 6:**
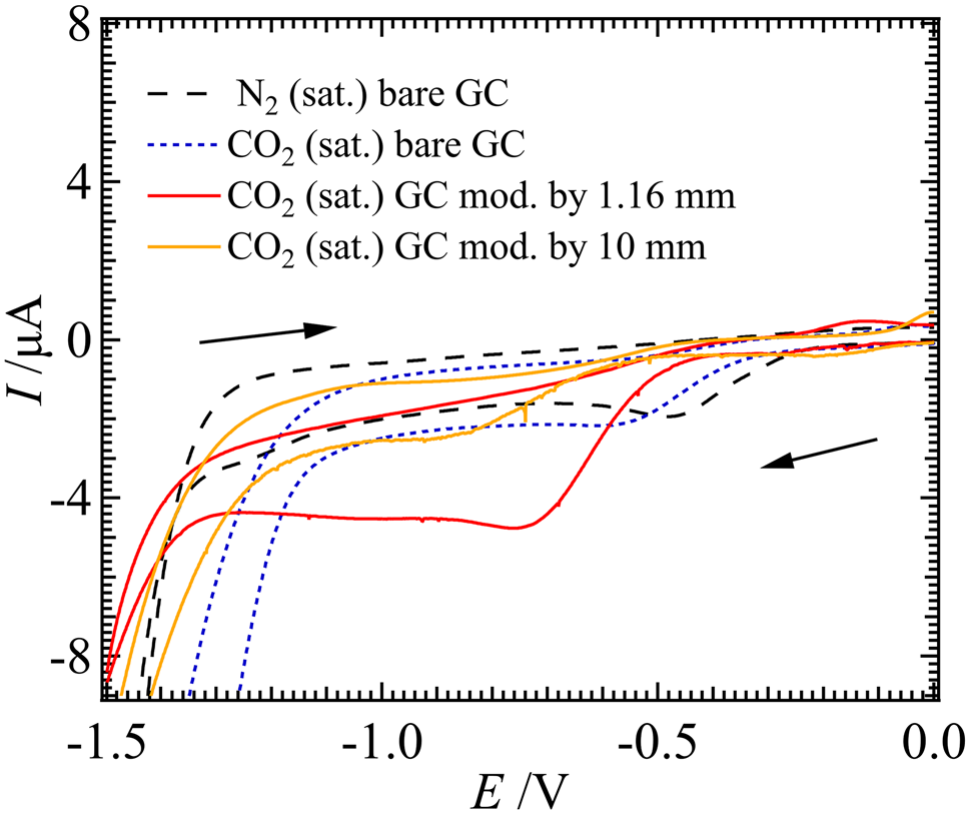
CVs recorded at a ~ 4 mm glassy carbon (GC) electrode immersed in a 0.1 M NaHCO_3_ aqueous solution without (bare) and with (modified) a layer of Cu NP/poly-TT film deposited on the surface. Films were electrogenerated at the 1.16 or 10 mm ITIES using Cells 1 or 2, as indicated inset, with [TT] = 20 mM and [CuSO_4_] = 5 mM, after 25 CV cycles at *v* = 0.020 V s^–1^. CVs were recorded in 3-electrode mode using a Ag/AgCl reference (Dek Research) and Pt wire counter electrode at 0.050 V s^–1^.

Preliminary electrocatalysis results were obtained by modifying the surface of a glassy carbon (GC) electrode with a layer of Cu NP/poly-TT film and using 0.1 M NaHCO_3_(aq) as supporting electrolyte. Figure 6 shows CVs recorded at a bare and modified electrode; whereby, the solution was purged with either N_2_ or CO_2_ gas for ~ 15 min prior to polarization.

At a bare GC electrode, and in the N_2_ saturated case, the cathodic peak at roughly – 0.45 V (vs. Ag/AgCl) is likely H^+^ reduction. However, this cathodic signal experiences a shift in the onset potential to – 0.57 V when purged with CO_2_ but maintains the same current intensity. The GC electrode modified with the Cu NP/poly-TT film electrosynthesized at a 1.16 mm ITIES shows a greater than 2 × CO_2_ reduction current at 0.75 V (vs. Ag/AgCl). To modify the GC electrode with a Cu NP/poly-TT composite at a 10 mm ITIES, the GC plug was dipped into the electrolytic cell after the 25 CV cycles were performed. Afterwards, the *i-V* response shown in Fig. 6, yellow trace, demonstrated a further shift in the overpotential towards more negative potentials with no increase in the peak current; thus, electrocatalysis is likely suppressed in this instance. Modification of the interface with a single deposit from the 25 µm diameter interface resulted in no significant change relative to the bare GC electrode (data not shown).

**Figure 7:**
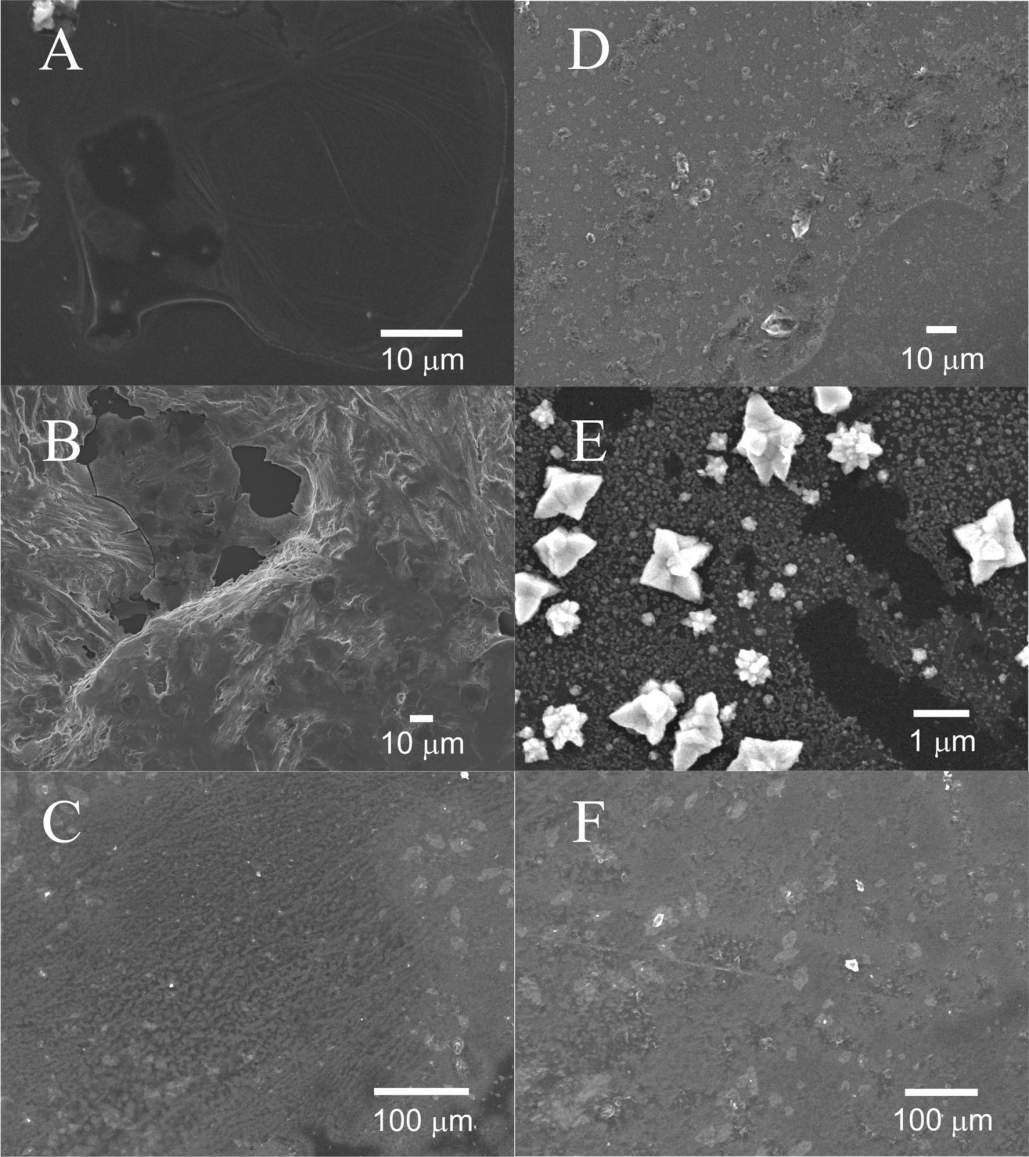
SEM micrographs of Cu NP/poly-TT film deposited on a glassy carbon (GC) electrode before (left-hand side) and after (right-hand side) CV electrocatalysis as shown in Fig. 6. The top (**A**,**D**), middle (**B**,**E**), and bottom (**C**,**F**) rows were films generated at a 25 µm, 1.16 mm, and 10 mm diameter ITIES, respectively. Scale is indicated inset.

Figure 7 depicts SEM images of the GC electrode surface modified with films generated at the 25 µm (A, D), 1.16 mm (B, E), and 10 mm (C, F) diameter interfaces before (left-hand side) and after (right-hand side) one CV cycle; additionally, using ImageJ software the GC surface coverage was estimated to be 0.3, 9.9, and 62.5%, respectively. The film electrosynthesized at the 25 µm ITIES is smooth and shows evidence of folding with Cu NPs distributed along creases in the film. It is hypothesized that the film quickly occludes the ITIES and new polymeric growth pushes the film into the aqueous side of the interface generating these folds. The Cu NP are likely concentrated towards the bottom of these folds, adjacent to the ITIES. Advanced, high-resolution optical methods will be needed to monitor film growth at the micro-ITIES in situ; however, this will be the focus of future work.

Films developed at the two large ITIES were smooth with a relatively even distribution of Cu NPs and no evidence of folding. Therefore, this phenomenon is likely owing to geometric confinement of the growing polymer film within the micropipette tip.

After electrocatalysis, the Cu NPs in the films generated at the 25 µm and 1.16 mm interfaces showed large changes in NP morphology which have grown by orders of magnitude; therefore, the thin, polymer network in these cases is insufficient to protect them against aggregation/agglomeration. The film created at the 10 mm ITIES showed little change; however, further experimentation is required.

While preliminary, these results are promising. Future work will focus on controlling Cu NP and polymer film morphology while tracking any changes the nanocomposite experiences during electrocatalysis, as well as detailed product analysis.

## Conclusions

The successful application of a micro-ITIES towards electrodeless synthesis of Cu NP/poly-TT has been demonstrated and compared to films generated at a large (mm scale) ITIES. At [TT] = 20 mM a well resolved electron transfer wave was observed at the micro-ITIES. However, the large interfaces showed a more complex CV profile with an irreversible electron transfer wave at high, positive potentials and a reversible signal towards the negative end. The latter is likely anion adsorption/exchange at the liquid|solid|liquid interfaces and agrees well with recent results by Scanlon’s group^4^. Impedance data confirm that the nanocomposite film forms early through large changes in *R*_CT_ and *R*_*C*_; moreover, increasing [TT] improves film formation while decreasing the median Cu NP size to < 2 nm.

Interestingly, while no electron transfer signal was observed at the micro-ITIES a low [TT], a film was electrogenerated and imaged using SEM. These data show that simply by probing the edge of the PPW one can facilitate electrodeless synthesis of the nanocomposite and avoid overoxidation of the polymer network.

Preliminary voltammetric results at a GC electrode modified with the Cu NP/poly-TT film electrogenerated at a 1.16 mm diameter interface elicited a > 2 × enhancement in the electrocatalytic CO_2_ reduction current versus an unmodified electrode. However, this film underwent large changes in NP morphology. While a tentative first step, these results are indicators that these films are promising alternative electrode materials for carbon capture; however, more optimization of nanocomposite electrosynthesis is necessary.

## Supplementary Information

Supplementary Information.

## Data Availability

All data is available upon request to T.J.S., tstockmann@mun.ca.
