# Supplementary material for: Simultaneous electro-generation/polymerization of Cu nanocluster embedded conductive poly(2,2′:5′,2′′-terthiophene) films at micro and macro liquid/liquid interfaces
Source: Sci Rep. 2023 Jan 21;13:1201. doi: 10.1038/s41598-023-28391-9 (PMC9867727; doi:10.1038/s41598-023-28391-9)
Supplement: Supplementary file 1 — Supplementary Information. [file 41598_2023_28391_MOESM1_ESM.pdf]

Supporting Information for: Simultaneous electro-generation/polymerization of Cu nanocluster embedded conductive poly(2,2′:5′,2″-terthiophene) films at micro and macro liquid/liquid interfaces

*Reza Moshrefi, Hanna Przybyła, and Talia Jane Stockmann**

Memorial University of Newfoundland, Chemistry Department, Core Science Facility, 45 Artic Ave., St. John's, NL Canada A1C 5S7
*Email: tstockmann@mun.ca (T.J.S.)

Table of Contents

1. Figure S1: CVs at a large ITIES, as well as SEM imaging and EDX analysis.
2. Figure S2: Histogram of Cu NPs generated at a micro-ITIES with 10 mM TT in DCE.
3. Figure S3: TEM micrographs and histrgrams of Cu NP sizes taken of Cu NP/poly-TT film electrogenerated at a 1.16 (**A, B**) and 10 mm (**C, D**) deposited on Au 200 mesh lacy carbon/ultra-thin film TEM grids. Cu NP sizes were measured using ImageJ software.
4. Figure S4: Photograph taken after the 1.16 mm diameter ITIES capillary was carefully removed from the organic phase. The thin, nanocomposite film can be seen covering the surface of the aqueous droplet.
5. Thermodynamics of Interfacial Electron Transfer
6. Micropipette/cell cleaning procedure.
7. References

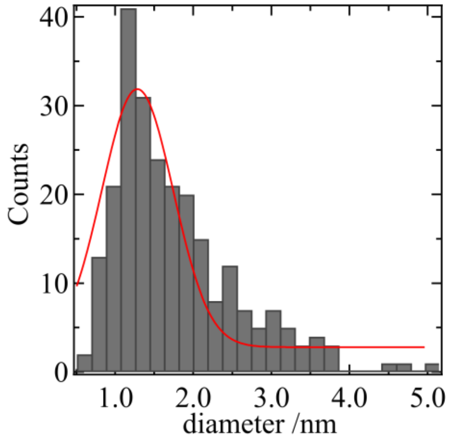

Figure S1: Histogram of Cu NPs sizes embedded in poly-TT after 25 CV scans using Cell 1 with [TT] = 10 mM in DCE.

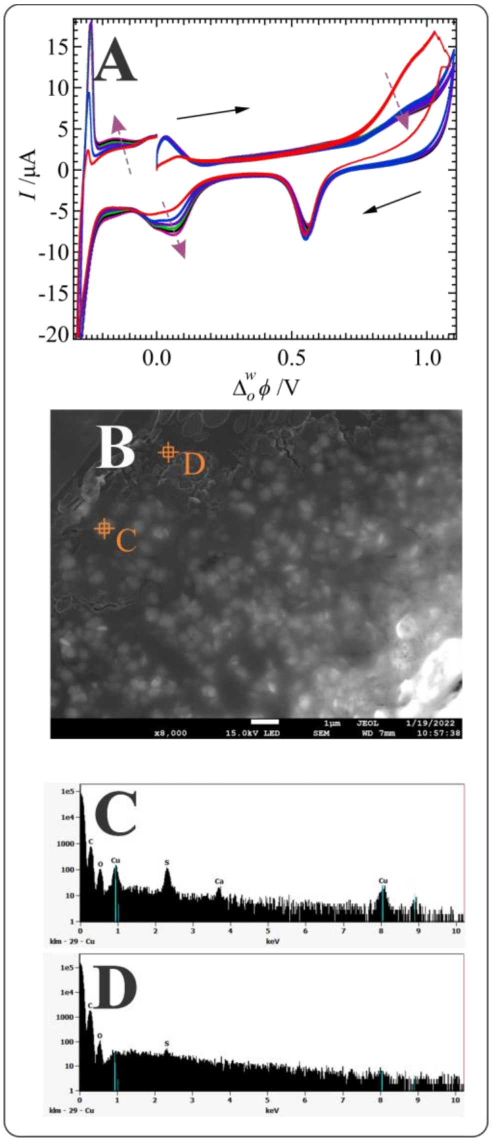

Figure S2: (**A**) 25 CVs recorded in succession using Cell 2 at a large, 10 mm diameter ITIES with [$CuSO_4$] = 1 mM, [TT] = 5 mM, and a scan rate of 0.020 V s^{-1}. The first and every subsequent 5th scan are displayed. Black arrows indicate scan direction, while dashed, purple arrows the evolution of current signals with each CV cycle. (**B**) SEM micrograph of the Cu NP/poly-TT film extracted from the large ITIES cell after the CV experiments shown in A and deposited on a glass slide. (**C**) and (**D**) are energy dispersive x-ray (EDX) spectra of the corresponding points indicated in B.

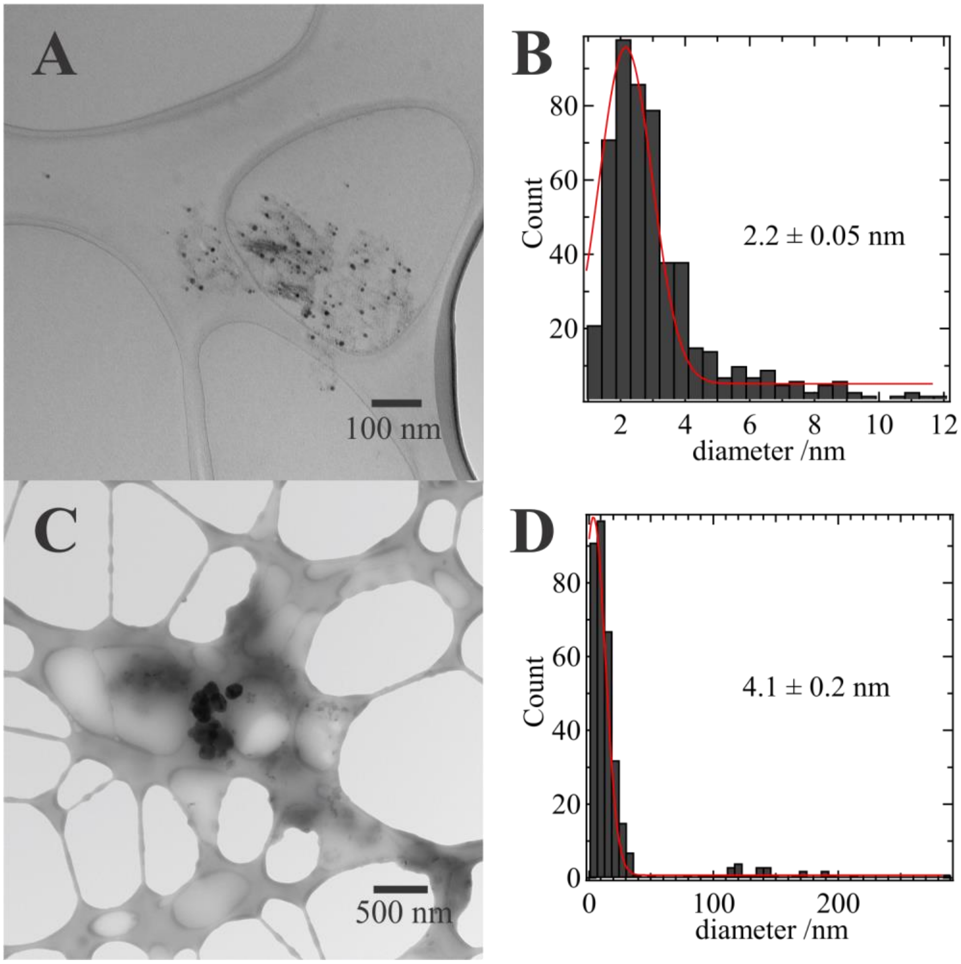

Figure S3: TEM micrographs and histrgrams of Cu NP sizes taken of Cu NP/poly-TT film electrogenerated at a 1.16 (**A, B**) and 10 mm (**C, D**) deposited on Au 200 mesh lacy carbon/ultra-thin film TEM grids. Cu NP sizes were measured using ImageJ software.

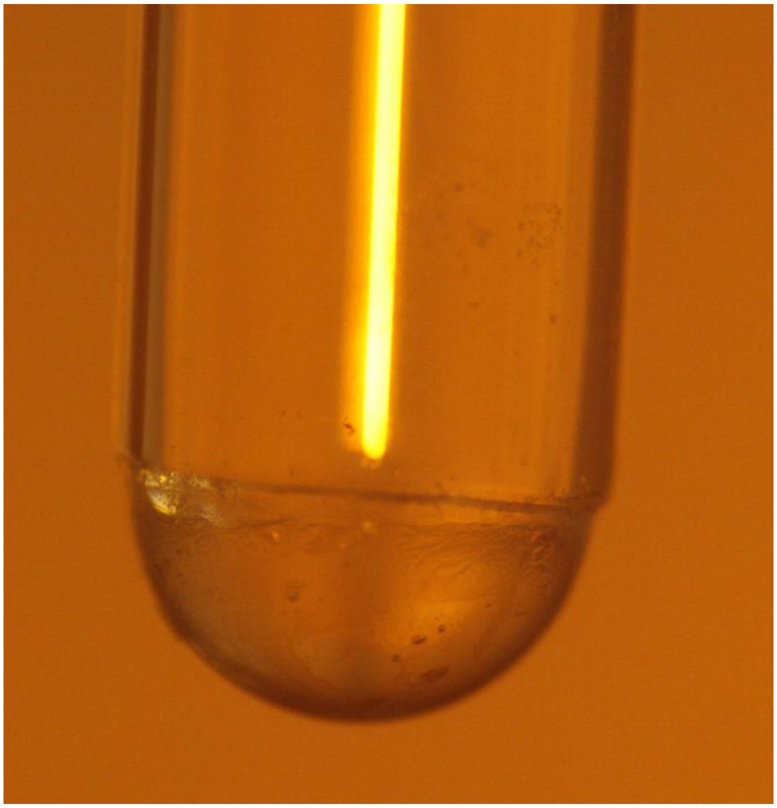

Figure S4: Photograph taken after the 1.16 mm diameter ITIES capillary was carefully removed from the organic phase. The thin, nanocomposite film can be seen covering the surface of the aqueous droplet.

Thermodynamics of Interfacial Electron Transfer

Using the fundamental equations for heterogeneous electron transfer at liquid|liquid interfaces described by Johans *et al.*[1] and others,[2] one can develop the following from general chemical reaction given in equation 5 of the main text as follows,

$$\Delta_o^w\phi_{\mathrm{ET}} = E^{o',\mathrm{DCE}}_{\mathrm{TT^{+\bullet}}/\mathrm{TT}} - E^{o',\mathrm{H_2O}}_{\mathrm{Cu(II)}/\mathrm{Cu}} - \frac{RT}{n_{\mathrm{TT^{+\bullet}}/\mathrm{TT}}\, n_{\mathrm{Cu(II)}/\mathrm{Cu}}\, F}\ln\left(\left[\mathrm{Cu}^{2+}\right]\left[\mathrm{H_2\text{-}TT}\right]^2\left[\mathrm{OH}^-\right]^2\right)$$

$$\Delta_o^w\phi_{\mathrm{ET}} = E^{o',\mathrm{DCE}}_{\mathrm{TT^{+\bullet}}/\mathrm{TT}} - E^{o',\mathrm{H_2O}}_{\mathrm{Cu(II)}/\mathrm{Cu}} - \frac{RT}{2F}\ln\left(\left[\mathrm{Cu}^{2+}\right]\left[\mathrm{H_2\text{-}TT}\right]^2\right) - \frac{RT}{2F}\ln\left[\mathrm{OH}^-\right]^2 \qquad \text{(S1)}$$

$$\Delta_o^w\phi_{\mathrm{ET}} = E^{o',\mathrm{DCE}}_{\mathrm{TT^{+\bullet}}/\mathrm{TT}} - E^{o',\mathrm{H_2O}}_{\mathrm{Cu(II)}/\mathrm{Cu}} - \frac{RT}{2F}\ln\left(\left[\mathrm{Cu}^{2+}\right]\left[\mathrm{H_2\text{-}TT}\right]^2\right) - \frac{RT}{F}\ln\left[\mathrm{OH}^-\right]$$

Neglecting the 3rd term on the right-hand side for simplicity, one arrives at,

$$\Delta_o^w \phi_{ET} = E^{o',DCE}_{TT^{+\bullet}/TT} - E^{o',H_2O}_{Cu(II)/Cu} - \frac{RT}{F}\ln\left[OH^-\right]$$
$$\Delta_o^w \phi_{ET} \approx E^{o',DCE}_{TT^{+\bullet}/TT} - E^{o',H_2O}_{Cu(II)/Cu} - \left(0.059\ V\right)\log\left[OH^-\right]$$
$$\Delta_o^w \phi_{ET} \approx E^{o',DCE}_{TT^{+\bullet}/TT} - E^{o',H_2O}_{Cu(II)/Cu} + \left(0.059\ V\right)pOH \qquad (S2)$$
$$\Delta_o^w \phi_{ET} \approx E^{o',DCE}_{TT^{+\bullet}/TT} - E^{o',H_2O}_{Cu(II)/Cu} + \left(0.059\ V\right)\left(14 - pH\right)$$

Micropipette/Cell cleaning procedure

All capillaries and glass electrolytic cells were cleaned extensively before use to prevent contamination between experiments. The cleaning procedure was as follows:

1. Rinse the capillary (or electrochemical cell) with DI water.

   Note: capillaries were rinsed using a syringe equipped with a flexible needle (MicroFil 28G, World Precision Instruments) and the inner solution was removed from the capillary using a small diameter tubing with one end attached to the house-air or N_2 line and the other affixed by hand to the micropipette end of the capillary, while holding a Kim-wipe at the open end of the capillary to catch the cleaning solution. Use caution as only a weak air/N_2 flow is needed. During sonication, capillaries were suspended in a 20 mL scintillation vial filled with DI water by drilling a hole in the plastic cap large enough to hold the capillary in place. Multiple holes were drilled in the cap and several capillaries were suspended in this way, in one scintillation vial at a time.

2. Fill the rinsed capillary/cell with a solution of 0.1 M $KMnO_4$ in 0.2 M H_2SO_4 and leave it to stand overnight.
3. Empty the washing solution and rinse with DI water to remove any remaining $KMnO_4$ solution left inside.
4. Fill the capillary/cell with a cleaning solution composed of 1 mL H_2O_2 (30%), 1 mL H_2SO_4 (95%) in 500 mL of DI water (piranha solution) and sonicate for 10-20 min. It is important not to exceed 20 min of sonication when cleaning the large ITIES electrochemical cells, as the solution can etch the glass and cause leaking around the Pt embedded electrodes.
5. Dispose of the cleaning solution in an appropriate container and rinse the capillary with DI water using the syringe with flexible needle and compressed air/N_2.
6. Next, fill the capillary with DI water and sonicate for 20 mins.
7. Finally, rinse the cell with DI water and it is ready to use.

References

1 Johans, C., Lahtinen, R., Kontturi, K. & Schiffrin, D. J. Nucleation at liquid|liquid interfaces: electrodeposition without electrodes. *J. Electroanal. Chem.* **488**, 99-109, doi:https://doi.org/10.1016/S0022-0728(00)00185-6 (2000).

2 Méndez, M. A. *et al.* Molecular electrocatalysis at soft interfaces. *Phys. Chem. Chem. Phys.* **12**, 15163-15171, doi:10.1039/c0cp00590h (2010).
